# The Use of Organic Rankine Cycles for Recovering the Heat Lost in the Compression Area of a Cryogenic Air Separation Unit

**DOI:** 10.3390/e24060748

**Published:** 2022-05-24

**Authors:** Claudia Ionita, Sorin Bucsa, Alexandru Serban, Catalina Dobre, Alexandru Dobrovicescu

**Affiliations:** Department of Engineering Thermodynamics, University Politehnica of Bucharest, 060042 Bucharest, Romania; claudia.ionita@upb.ro (C.I.); sobucsa@yahoo.com (S.B.); alexandru.serban@upb.ro (A.S.)

**Keywords:** exergetic analysis, exergetic destruction, organic fluid, internal heat exchanger

## Abstract

The use of organic Rankine cycles (ORCs) is a viable solution for the recovery of waste heat. For an air separation unit (ASU) with a production of ${\dot{V}}_{O_{2}}=58300^{}\left[m_{N}^{3}/h\right]$ operating in Romania, the value of utilization of the heat transferred to the cooling system of the compression area represents 21% of the global system electrical energy input. To recover this thermal energy and transform it into mechanical energy, an ORC system was proposed. To maximize the production of mechanical power, an exergy analysis was performed. Exergy analysis was used to choose the most suitable organic fluid and find the optimum constructive structure of the Rankine cycle. The calculation of the exergy destruction in the key apparatuses of the system allowed investigation into the optimization search procedure. The large exergy destruction in the liquid preheater suggested the decrease in the temperature difference in this part of the evaporator by increasing the inlet temperature of the liquid; and an internal recuperative heat exchanger was used for this purpose. When permitted, the overheating of the vapors also reduced the temperature difference between the heat source and the organic fluid during the heat transfer process. The analysis was comparatively performed for several organic fluids such as R-245fa, R123, n-pentane and R717. The use of ammonia, that offered the possibility of superheating the vapors at the turbine inlet, brought a gain of mechanical power corresponding to 6% economy in the electrical energy input of the global plant.

## 1. Introduction

When there is a heat source at a higher temperature than the environment, there is a possibility of transforming it into mechanical energy with the help of a power cycle. This also occurs if the cold source of the power cycle is at a lower temperature than the environment, in which case, some of the mechanical energy consumed to obtain the low temperature cold source can be recovered [1].

Power cycles can be open, in which the air represents the working fluid that is introduced in the cycle at the parameters of the environment, or closed, in which the working fluid has a different composition to that of the environment [1]. In the case of closed Rankine cycles, the advantage of achieving heat exchanges with hot and cold sources at almost constant temperatures, leads to an increase in the energy efficiency of the cycle, bringing it closer to that of the Carnot cycle. To this, the advantage of increasing the fluid pressure in the liquid phase is added; the increase in pressure is achieved by a practically insignificant consumption of mechanical energy in the pump [1].

The use of organic fluids in Rankine cycles leads to simple functional schemes in which, for fluids with complex molecular composition, the appearance of liquid is avoided during the expansion process in the expander. The use of high molecular weight organic fluids has the effect of reducing the enthalpy drop in the expander which leads to a decrease in its number of stages or to a decrease in the peripheral speed at the exit of the turbine [2,3,4]. The choice of organic fluids with higher critical temperatures but appropriate to those of the heat sources leads to condensation pressures higher than the ambient pressure, thus eliminating the special measures for sealing the condenser [5,6,7,8,9].

In the analysis of the recovery of limited heat sources at moderate heat levels, the research focused on optimizing the operation and construction of ORC schemes looking for investigative methods to highlight and quantify the negative effects of malfunctions associated with internal processes.

The technique of investigation chosen by most researchers is the exergetic method based on the union between the First and Second Laws of Thermodynamics [10,11,12]. An energy and exergy analysis of the integration of an ORC in a solid oxide fuel cell (SOFC) system was performed by Ahmadi M.H. et al. [13]. The ORC used, as heat sink, a stream of LNG on its way to regasification, and as heat source it used the flue gas generated by the afterburner of the SOFC that later is expanded in a gas turbine. The work calculated the exergetic performances of the key parts of the system, offering information about opportunities of improvement. Effatpanah S.K. et al. [14] conducted an exergoeconomic analysis of the recovery of heat waste from a biomass combustion process with the use of ORC. The analysis revealed that the most destructive parts of the ORC were the evaporator and the turbine. The exergoeconomic analysis enabled the determination of the monetary cost of the output power and the capital investment amortization rate of the key apparatuses. Shamoushaki M. et al. [15] conducted a comprehensive analysis and optimization of different configurations of power cycles. The method of analysis combined the energetic and exergoeconomic balances. The study pointed out the advantages of using a simple ORC in terms of the power generation cost and pay-back period. Feng Y. et al. [16] used the concept of entropy generation and entransy dissipation [17,18,19] to analyze a basic ORC operating with mixtures of working fluids. They studied the effect of the temperature glides in the evaporator and condenser on the irreversibility of heat transfer under a finite temperature difference. They found a minimum entropy generation in the evaporator for a mixture of R245fa and pentane, while in other pieces of equipment, other pairs of organic fluids reported minimum entropy generation.

Liu C. et al. [20] studied, based on the Second Law of Thermodynamics, the influence of the evaporation temperature on the performance of subcritical ORC. The analysis considered 28 working fluids used in an ORC to recover waste heat. They studied the influence of the critical temperatures of the working fluids on the choice of the optimum temperature of evaporation. The work reported recommendations for the temperature difference between the critical temperature of the organic fluid and the temperature of the waste heat source for an optimum evaporation temperature that led to a maximum Second Law efficiency.

In [21], Ho Yong Lee and Kyoung Hoon Kim discussed the use of LNG as a heat sink in a combined power cycle composed of an ORC and an LNG power generation by direct expansion. The analysis considered different organic fluids and accounted for the influence of the critical temperature of the working fluids and inlet pressure in the turbine on the mechanical power and cycle efficiency.

An analysis of a combined heat and power engine fueled with biogas and subcritical or supercritical organic Rankine cycles was performed by Koc Y. et al. [22]. The study, based on exergy analysis, sought to determine the best operational conditions to achieve the maximum mechanical work from the recovered heat carried by the flue gas of the thermal engine. They reported the highest exergy destruction in the evaporator. The paper reached the conclusion that the best performance was achieved by the subcritical ORC with a regenerative heat exchanger.

Exergetic analysis was used by Liu G. et al. [23] to determine the best functional parameters for the efficient operation of the power system using two organic Rankine cycles running separately at two different heating temperatures. Several organic fluids were comparatively studied. The mass flow rate of the hot carrying fluid was split into two branches that fed the evaporators of the two ORC stages. The splitting ratio of the mass flow rate was a decisional parameter in the optimization search procedure. The work determined the organic fluids and their splitting ratio for supplying both evaporators of the two-stage system, suitable for different heat source temperatures.

The optimization of an organic Rankine cycle and the nature of the fluids’ influence on the performance of the system were studied by Fergani Z. et al. [24]. The analysis was based on the exergy concept, and the optimum search appealed for multi-objective optimization. The exergoeconomic model offered economical correlations for estimating the investment cost of the key pieces of equipment. The exergetic efficiency and the unitary monetary cost of the product of the system were calculated at the variation of the composition of the zeotropic mixture. The exergoeconomic analysis determined the most cost destructive processes, and the most expensive apparatuses. The conclusion was that, compared with operating with their pure components, the use of zeotropic mixtures in ORC brought an increase in the performance of the cycle. Dorosz P. et al. [25] coupled an LNG system with power delivery by direct expansion with the condenser of an ORC, and recovered a part of the necessary heat for regasification. The performances of the cycle were ranked based on exergetic efficiency, and the decisional parameters were the pumping pressures in the LNG and ORC systems.

The present paper, based on exergetic analysis, aims to reveal the malfunctions associated with the working processes, offering a strategy for functional and structural optimization. The magnitude and location of exergy destructions will guide the search for the most efficient operating regime and system configuration.

## 2. Potential for Recovery of Heat from the Cryogenic Air Separation Unit

**Figure 1:**
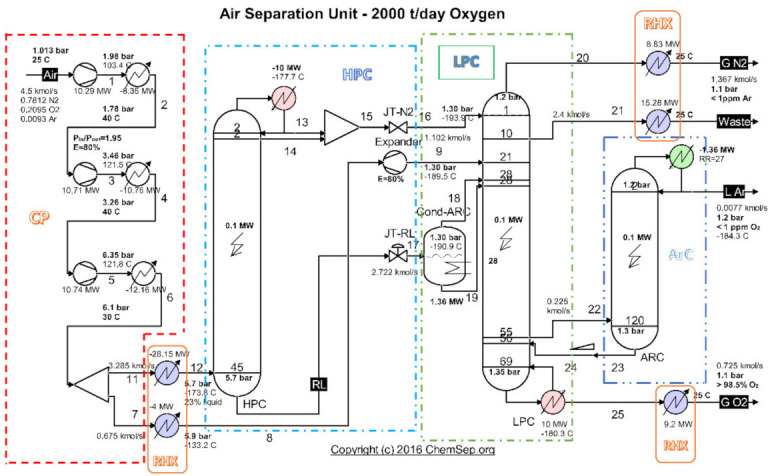
Schematic of the cryogenic air separation installation–functional areas. Source: Harry Kooijman (2006) chemsep.org [26].

An analysis of the evacuated heat recovery potential was performed for the cryogenic air separation unit (ASU) (Figure 1) operating on the platform of the Galati Iron and Steel Works, Romania [26].

Highlighting as final products of the installation the currents of oxygen gas, nitrogen gas and liquid argon (Figure 1 and Table 1), and as resource (fuel) the electricity consumed to drive the compressors (Figure 1 and Table 2), the exergetic analysis of the installation showed a global exergetic performance coefficient of 14% (Equation (1)) [26].

The exergetic performance coefficient of the whole system is:

(1) $$COP_{ex}=\frac{P}{F}=\frac{\dot{E}x_{GN_{2}}^{TOT}+\dot{E}x_{LAr}^{TOT}+\dot{E}x_{GO_{2}}^{TOT}}{{\dot{E}}_{el}}=\frac{4485.2}{31755}=0.14.$$

**Figure 2:**
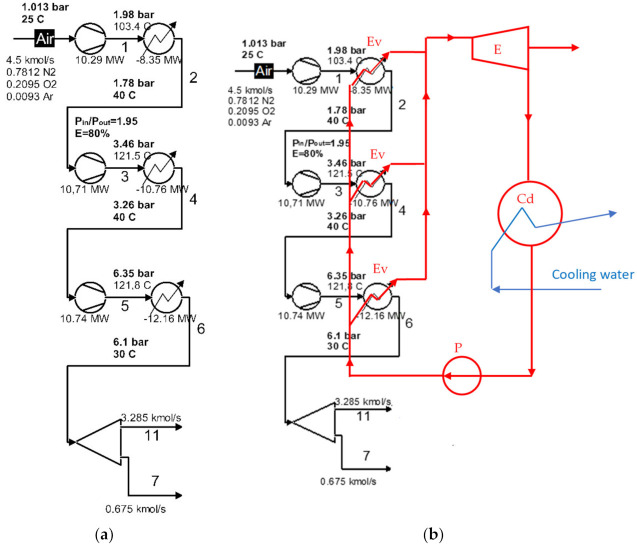
Schematic of the compression area of the cryogenic air separation unit: (**a**) the initial compression area; (**b**) schematic of the use of ORC for intermediate and final cooling of compressors.

The exergetic analysis of the installation demonstrated that the compression stage (Figure 2a) represented an area with a major destruction of exergy (consumption of electricity).

Table 3 shows the exergy destructions due to the irreversibility of the compression processes in the compression stages, and Table 4 shows the exergy losses with the heat transferred in the intermediate coolers and the final of the compression stages [26].

The sum of exergy losses and destruction in the compression zone is:

(2) $${\dot{I}}_{Z,cp}={\dot{I}}_{cp}+{\dot{L}}_{Q}=4.958+6.781=11.739\;MW.$$

The share of exergy losses and destruction in the compression zone in the fuel consumption of the global installation is:

(3) $$\psi_{cp}=\frac{{\dot{I}}_{Z,cp}}{\left|{\dot{W}}_{1}\right|+\left|{\dot{W}}_{2}\right|+\left|{\dot{W}}_{3}\right|}=\frac{11.739}{31.75}=0.37.$$

It was observed that 37% of the electricity consumption of the global installation was dissipated in the irreversible processes of the compression zone (Equations (2) and (3)) [26].

Of this exergy consumption, more than half (21% of global consumption) represented the exergy loss with the heat transferred to the environment (Equation (4)) [26].

(4) $$\psi_{Q,cp}=\frac{{\dot{L}}_{Q}}{\left|{\dot{W}}_{1}\right|+\left|{\dot{W}}_{2}\right|+\left|{\dot{W}}_{3}\right|}=\frac{6.781}{31.75}=0.21.$$

If the reduction in the exergy destruction associated with the compression process is largely impossible to counteract due to the limited isentropic efficiency of the compressors, conversely, the heat dissipated in the cooling system of the compressors is a source of recoverable energy with a major consequence for increasing the efficiency of the global system.

In the case of an installation of the complexity and quantity of product of the size of the cryogenic air separation unit of the Galati Iron and Steel Works, the heat recovery and its transformation into electricity will be a solution of interest.

Due to the relatively low average temperature level (70–80 °C) of the heat discharged by the compressor cooling system, a viable solution would be to transform this heat into mechanical work (electricity) using a Rankine cycle running on organic fluids.

Instead of using cooling water in the intermediate coolers and the final cooler, an organic fluid is proposed to be used, which is also the working fluid of the Rankine cycle.

## 3. The Recovery of the Heat Discharged from the Compressors Cooling System Using a Rankine Cycle with Organic Fluids

A flow chart of the heat recovery from the compressors cooling system of the cryogenic air separation unit using a Rankine cycle with organic fluids (ORC) is shown in Figure 2b.

According to Figure 2b, the organic fluid was divided into three streams that receive, in parallel, the heat from the air coolers of the compression stage. The distribution of the mass flow of the organic fluid was conducted in proportion to the thermal load of each chiller, to ensure that each of the streams of organic fluid were heated to the same temperature. After the heat recovery, the gaseous organic fluid expanded into the expander of the ORC system, eventually producing electricity.

The inlet and outlet temperatures of the hot fluid (compressed air) in the evaporator are shown in Table 5 and Figure 2.

The potential for recovery of the heat discharged by the compression stage of the ASU is shown in Table 5.

### 3.1. Rankine Cycles with Organic Fluids

**Figure 3:**
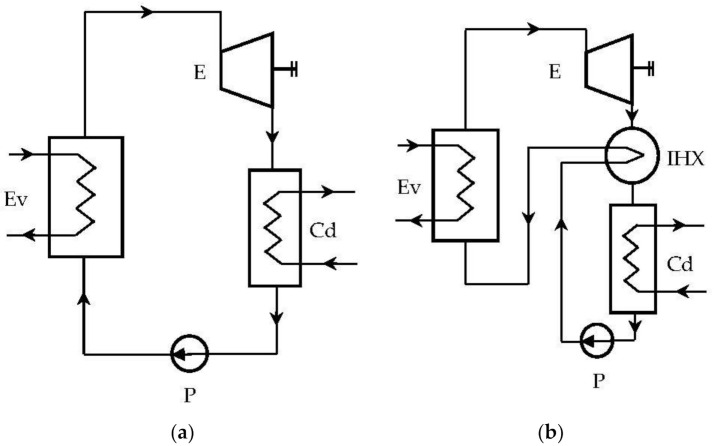
Rankine system with organic fluids: (**a**) ORC system without internal heat exchanger (IHX); (**b**) ORC system with IHX.

In the case of ORC cycles operating “dry”, a recuperative (internal) heat exchanger can be provided, which, based on the high thermal potential of the expander outlet, can preheat the liquid (Figure 3b).

### 3.2. Organic Fluids

An important feature of organic fluids is the slope of the dry saturated vapor curve in the T-s diagram, a slope that imposes the state of the fluid at the exit of the expander after adiabatic expansion.

Depending on the slope of the vapor saturation curve, organic fluids fall into three categories:

(a) “Wet” fluids that have a negative vapor saturation curve slope-example: NH_3_, R152a, R134a;
(b) “Dry” fluids that have a positive vapor saturation slope-example: n-pentane, R236fa, R141b;
(c) “Isentropic” fluids with an infinite slope, the vapor saturation curve being practically perpendicular to the entropy axis-example: R-245fa, R-123.

Cycles running on dry or isentropic fluids were expected to have higher efficiencies than those using wet fluids due to the absence, in the case of the former, of the condensation process it accompanied; and in the case of wet fluids, expansion in the expander.

## 4. Thermodynamic Analysis of ORC Cycles

The thermodynamic analysis aimed to model the operation of ORC cycles with different working agents and to simulate their behavior in conditions of coupling with the coolers of the compression area of the cryogenic air separation unit.

The selected organic fluids belong to all the categories discussed [27,28,29,30]:

(1) Wet fluids: NH_3_;
(2) Isentropic fluids: R123, R245fa (single component fluids with excellent environmental properties that replace Freon-11 which is being phased-out);
(3) Dry fluids: n-pentane.

### 4.1. ORC Cycle with Non-Negative Vapor Saturation Curve Slope without Vapor Overheating at the Inlet to the Expander and without Internal Recuperator Heat Exchanger

**Figure 4:**
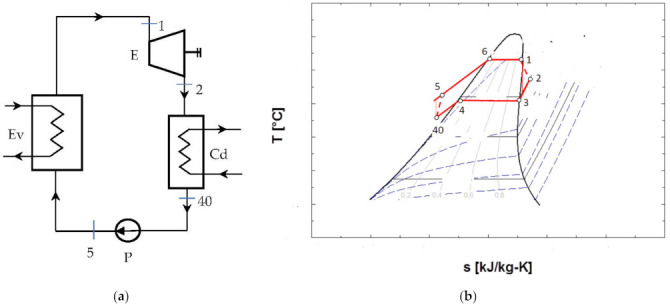
ORC system without overheating of the vapors at the entrance to the expander and without internal recuperative heat exchanger: (**a**) flow chart; (**b**) representation in the T-s diagram of the ORC cycle.

The schematic of the ORC system and the representation of the cycle in the diagram T-s, are shown in Figure 4.

Figure 4b shows that the cycle, in the chosen functional variant, worked with the subcooling of the condensed liquid.

### 4.2. ORC Cycle with Non-Negative Vapor Saturation Curve Slope, without Overheating of Vapors at the Inlet to the Expander, with Internal Heat Exchanger

**Figure 5:**
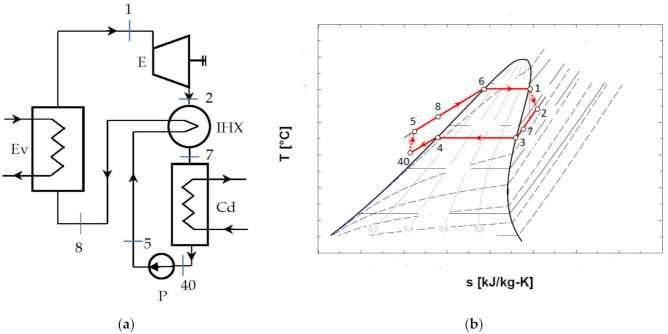
ORC system without overheating and with internal heat exchanger recuperator: (**a**) schematic of the system; (**b**) representation in the T-s diagram of the ORC cycle.

The diagram of the ORC system with internal heat exchanger recuperator, and the representation of the cycle in the diagram T-s, are presented in Figure 5.

### 4.3. ORC Cycle with Non-Negative Vapor Saturation Curve Slope with Vapor Overheating at the Inlet to the Expander and with Internal Heat Exchanger

**Figure 6:**
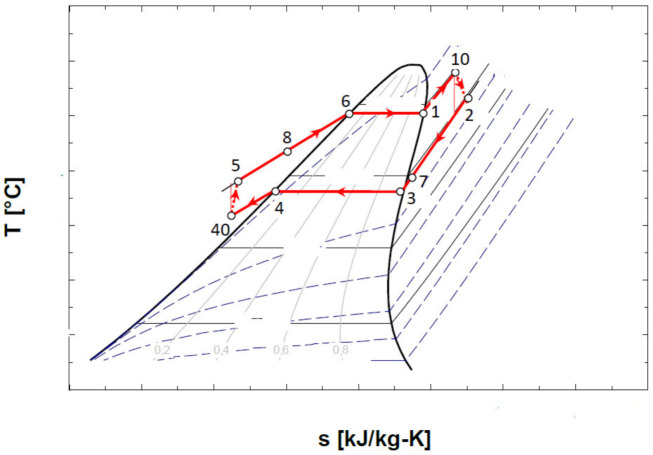
Representation in the T-s diagram of the ORC cycle with the overheating of the vapors at the entrance to the expander and with the internal heat exchanger recuperator.

The representation in the T-s diagram of the ORC cycle with the overheating of the vapors before entering the expander and with the internal recuperative heat exchanger, is shown in Figure 6.

### 4.4. Mathematical Modeling and Energy Analysis of the ORC Cycle

The design of the mathematical model was conducted for the scheme without an internal heat exchanger and without overheating of the vapors at the exit of the evaporator (Figure 4); for the other schemes, the main elements such as the choice of vaporization and condensation temperatures remained unchanged.

**Figure 7:**
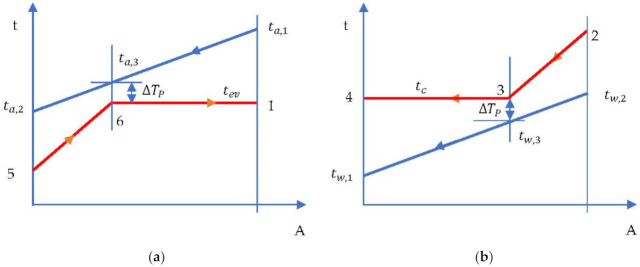
ORC cycle without overheating and without internal heat exchanger. Temperature-area of heat transfer (t-A) diagram: (**a**) evaporator; (**b**) condenser.

- The evaporation temperature was imposed by the minimum temperature difference in the evaporator (temperature difference at Pinch) which occurred at the end of heating the liquid in the evaporator and the beginning of evaporation (Figure 7a).

***Given:***

☑ Air temperatures $t_{a,1}$ and $t_{a,2}$ at the inlet and outlet of the cooler, respectively;

***And designer’s choices:***

☑ The nature of the ORC fluid;
☑ The minimum temperature difference $\Delta T_{P}$;

***Then:***

☑ The vaporization temperature of the ORC fluid is determined.
☑ From the energy balance of the vaporization zone the temperature, t_ev_, results.

(5) $${\dot{Q}}_{6-1}={\dot{Q}}_{a,1-3}\to \to {\dot{m}}_{a}c_{p}\left(t_{a,1}-t_{a,3}\right)={\dot{m}}_{ORC}\left[h_{1}\left(t_{ev},x=1\right)-h_{6}\left(t_{ev},x=0\right)\right]$$

(6) $$t_{ev}=t_{a,3}-\Delta T_{p}$$

(7) $${\dot{m}}_{a}c_{p}\left(t_{a,1}-t_{a,2}\right)={\dot{m}}_{ORC}\left[h_{1}\left(t_{ev},x=1\right)-h_{5}\left(p_{ev}\right)\right]$$

(8) $$p_{ev}=p\left(t_{ev}\right).$$

The system consisting of Equations (5)–(8) has four variables, namely, $t_{a,3},{\;t}_{\mathrm{ev}},\;{\dot{m}}_{ORC},{\;p}_{\mathrm{ev}}$, and is, therefore, determined. The enthalpy in state 5 is determined in a subsequent procedure when running the cycle.

- Condensation temperature is determined by the temperature of the cooling water and the minimum temperature difference in the condenser (temperature difference at Pinch $\Delta T_{P}$) (Figure 7b).

***Given:***

☑ Water temperature $t_{w,1}$ at the condenser inlet;

***And designer’s choices:***

☑ The increase in condenser cooling water temperature $\Delta T_{c}$;
☑ The minimum temperature difference ∆T_p_ in the condenser;

***Then:***

☑ The condensation temperature $t_{c}$ of the ORC fluid is determined.

The energy balance of the condenser is:

(9) $${\dot{Q}}_{w,1-3}={\dot{Q}}_{3-4}\to \to {\dot{m}}_{w}c_{w}\left(t_{w,3}-t_{w,1}\right)={\dot{m}}_{ORC}\left[h_{3}\left(t_{c},x=1\right)-h_{4}\left(t_{c},x=0\right)\right]$$

(10) $$t_{c}=t_{w,3}+\Delta T_{p}$$

(11) $${\dot{m}}_{w}c_{w}\left(t_{w,2}-t_{w,1}\right)={\dot{m}}_{ORC}\left[h_{2}\left(p_{c}\right)-h_{4}\left(t_{c,},x=0\right)\right]$$

(12) $$t_{w2}=t_{w1}+\Delta t_{w}$$

The four Equations (9)–(12) have four variables:${\;t}_{c},{\;m}_{w},{\;t}_{w,3},{\;t}_{w,2}$.

The expander output:

(13) $${\dot{W}}_{E}={\dot{m}}_{ORC}\left(h_{1}-h_{2}\right)$$

The pump input:

(14) $${\dot{W}}_{P}={\dot{m}}_{ORC}\left(h_{5}-h_{4}\right)$$

The net output of the ORC:

(15) $$\dot{W}={\dot{W}}_{E}-{\dot{W}}_{P}$$

The energetic COP of the ORC:

(16) $$COP_{en}=\frac{\dot{W}}{\;{\dot{Q}}_{\mathrm{av}}}$$

### 4.5. Exergetic Analysis of the ORC Cycle

To define an operational and constructive optimization strategy, the exergetic method was used, which identified the place and size of a malfunction in the system [31,32].

The exergetic balance equation for the cycle without overheating and without internal recuperator heat exchanger (Figure 4) is:

(17) $$\sum \dot{E}x_{Q}=\Delta \dot{E}x+\dot{W}+\sum \dot{I}$$

For the ORC cycle, $\Delta \dot{E}x=0$ and the rest of the terms of Equation (17) become:

(18) $$\sum \dot{E}x_{Q}=\dot{E}x_{Q}^{T_{1-5}}+\dot{E}x_{Q}^{T_{2-4}}=\dot{E}x_{Q}^{T_{1-5}}-\left|\dot{E}x_{Q}^{T_{2-4}}\right|$$

(19) $$\sum \dot{I}={\dot{I}}_{E}+{\dot{I}}_{p}$$

Substituting relationships (18) and (19) in the relation (17) we obtain:

(20) $$\dot{E}x_{Q}^{T_{1-5}}-\left|\dot{E}x_{Q}^{T_{2-4}}\right|=\;\dot{W}+\;{\dot{I}}_{E}+\;{\dot{I}}_{P}$$

Noting that the heat flow to the evaporator was obtained from the cooled compressed air stream, and considering the destruction of exergy due to the heat transfer at the finite temperature difference in the evaporator, the balance Equation (20) becomes:

(21) $$\dot{E}x_{Q}^{T_{a,1-2}}=\;\dot{W}+\left|\dot{E}x_{Q}^{T_{2-4}}\right|+\;{\dot{I}}_{\Delta T,\mathrm{ev}}+\;{\dot{I}}_{E}+{\dot{I}}_{P}$$

in which:

(22) $${\dot{I}}_{\Delta T,\mathrm{ev}}=\dot{E}x_{Q}^{T_{a,1-2}}-\dot{E}x_{Q}^{T_{1-5}}={\dot{Q}}_{a}T_{0}\frac{T_{a,1-2}-T_{1-5}}{T_{a,1-2}\cdot T_{1-5}}$$

(23) $${\dot{I}}_{E}={\dot{m}}_{ORC}T_{0}\left(s_{2}{-s}_{1}\right)$$

(24) $${\dot{I}}_{P}={\dot{m}}_{ORC}T_{0}\left(s_{5}{-s}_{4}\right)$$

(25) $$\dot{E}x_{Q}^{T_{a,1-2}}={\dot{Q}}_{a,1-2}\left(1-\frac{T_{0}}{T_{a,1-2}}\right)=\;{\dot{E}x}_{Q,\mathrm{av}}$$

where ${\dot{\mathrm{Ex}}}_{Q,\mathrm{av}}\;$ represents the exergy of the available heat carried by the air that is the fuel of the system.

The exergetic efficiency is:

(26) $$\eta_{ex}=\frac{\dot{W}}{\dot{E}x_{Q,av}^{}}$$

The share of an exergy destruction in the exergy of the heat available for recovery becomes:

(27) $$\psi =\frac{\dot{I}}{\dot{E}x_{Q,av}^{}}$$

## 5. Study of the Performance of the ORC Cycle Working with Different Organic Substances

The ORC system recovered the heat evacuated by the coolers of the compression area of the cryogenic air separation unit to transform it into mechanical (electrical) energy.

To correctly estimate the real performance of the system, an exergetic analysis was performed, which highlighted the functional areas with potential for improvement, which allowed the establishment of a strategy for the functional and constructive optimization of the ORC system. A simulation of the operation of the organic Rankine cycles was performed with the EES program [33].

In all studied cases, the isentropic efficiencies of the expanders and pumps were $\eta_{sE}=0.85$ and $\eta_{sP}=0.6$, respectively. The condensing temperature was $t_{c}=20\;{}^{\circ }C$, under the conditions in which the cooling water of the condenser before entering the condenser was cooled by means of the residual nitrogen discharged from the low-pressure distillation column. To ensure all the heat exergies were positive, the reference ambient temperature was chosen as $t_{0}=10\;{}^{\circ }C$.

To counteract the decrease in the temperature at the pinch point in the evaporator with the increase in the vaporization temperature, the condensed liquid was subcooled before the suction in the pump (Figure 4, Figure 5 and Figure 6).

### 5.1. Study of the Performance of the ORC Cycle Operating with R-245fa

The decisional parameter according to which the optimization study was conducted, was the evaporation temperature, $t_{ev}$.

In the case of R-245fa, the evaporation temperature varied in the range of $35-50\;{}^{\circ }C$.

#### 5.1.1. ORC System with R-245fa without Overheating and without Internal Recovery Heat Exchanger 

The schematic of the system is presented in Figure 4. The results of the mathematical modeling based on the exergetic analysis of the ORC operating with R-245fa, without overheating and without internal heat exchanger, are presented in Table 6.

The exergetic balance is represented by the exergetic efficiency $\eta_{ex}$ and the weights of the exergy destruction related to the exergy of the available heat to be recovered $\psi =\dot{I}/\dot{E}x_{Q.av}$.

**Figure 8:**
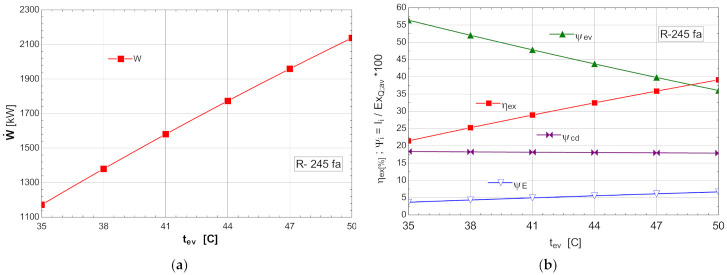
ORC without overheating and without internal heat exchanger: (**a**) mechanical power, depending on the variation of the evaporation temperature $t_{ev}$; (**b**) exergetic efficiency and weights of the exergy destruction relative to the exergy of the available recovered heat, depending on the variation of the evaporation temperature, $t_{ev}$.

The variations of the energetic and exergetic measures at the change of the evaporation temperature $t_{ev}$ are presented in Figure 8b.

As the evaporation temperature increased, the exergetic efficiency of the cycle rapidly increased (Figure 8b).

The exergy destructions with the highest weights corresponded to the processes in the vaporizer ($\psi_{ev}$) and condenser–subcooler ($\psi_{cd}$) (Figure 8b). Increasing the temperature of the organic fluid ($t_{ev}$) that absorbed the recovered heat, reduced the anergy of this transfer process.

The increase in the temperature of the ORC fluid that received the heat recovered from the cooling system of the ASU compression area could be achieved by recovering, in an internal heat exchanger, the heat of the vapors from the outlet of the expander when the temperature difference $\left(t_{2}-t_{5}\right)$ allowed (Table 6).

As a result of the reduction in the exergy destruction in the vaporizer with the increase in the vaporization temperature, the mechanical power ($\dot{W}$) produced by the ORC rapidly increased (Figure 8a).

#### 5.1.2. ORC System with R-245fa without Overheating with Internal Recovery Hear Exchanger 

The schematic of the system is presented in Figure 5.

**Figure 9:**
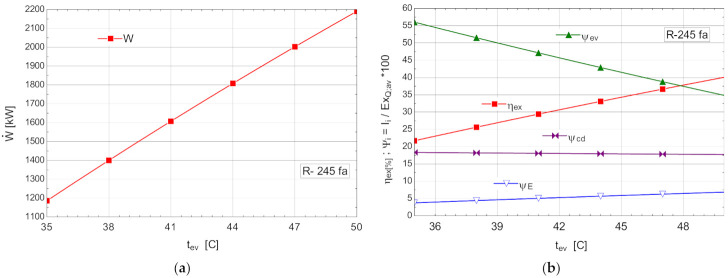
ORC with internal heat exchanger and without overheating: (**a**) mechanical power, $\dot{W}$, depending on the variation of the evaporation temperature, $t_{ev}$; (**b**) exergetic efficiency and weights of exergy destructions relative to the exergy of the recovered available heat, depending on the variation of the evaporation temperature, $t_{ev}$.

The high destruction of exergy in the vaporizer led to the introduction of an internal recuperative heat exchanger [34]. The primary goal was to increase the mechanical power under conditions of a steadily recovered heat flux. The influence of the variation of the evaporation temperature on the energetic and exergetic measures of the ORC with internal recuperative exchanger is presented in Figure 9.

The efficiency of the internal recuperative heat exchanger was estimated at $\varepsilon_{IHX}=0.8$.

The results of the mathematical modeling based on the exergetic analysis of the ORC operating with R-245fa, without overheating, with internal heat exchanger are presented in Table 7.

Compared with the scheme without the internal heat exchanger, the expected decrease in exergy destruction in the vaporizer was small, but considering the ORC mass flow rates, there was an increase in the mechanical power developed. Thermodynamically, the ORC with internal heat exchanger was more efficient than the one without the internal recuperator.

#### 5.1.3. ORC System with R-245fa with Overheating and Internal Recovery Heat Exchanger 

The schematic of the system is presented in Figure 6.

**Figure 10:**
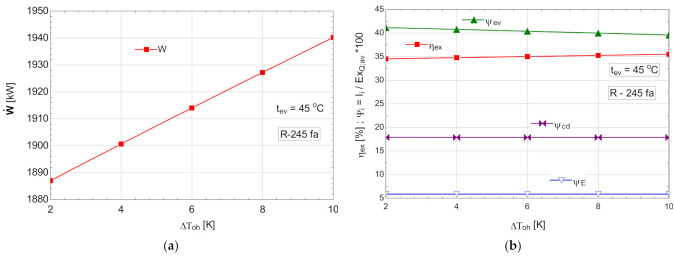
ORC with internal heat exchanger and overheating: (**a**) mechanical power, $\dot{W}$, depending on the degree of overheating, $\Delta T_{oh}$; (**b**) exergetic efficiency and weights of exergy destructions relative to the exergy of available recovered heat, depending on the variation of the degree of overheating, $\Delta T_{oh}$.

Starting from the most efficient variant characterized by the highest vaporization temperature that would allow the temperature difference prescribed at the pinch point to be ensured, an attempt to overheat the vapors above this temperature was conducted. For thermo-economic reasons imposed by the temperature difference at the pinch point, the temperature of evaporation was limited at 45 °C for all organic Rankine fluids. The results of the study with overheating for an evaporation temperature $t_{ev}=45^{o}C$ are presented in Table 8 and Figure 10.

As expected, the increase in the degree of overheating $\Delta T_{oh}$ led to a decrease in the exergy destruction of the evaporator-superheater in the conditions of maintaining a constant loss of exergy in the condenser (Figure 10b). The exergetic efficiency of the cycle increased (Figure 10b) in addition to the mechanical power output (Figure 10a).

Although the ORC flow rate decreased (Table 8), the developed mechanical power increased, which was a double positive effect.

To highlight the mechanism of reducing the destruction of exergy in the evaporator which led to the increase in the mechanical power developed by the system, the destruction of exergy was divided according to the areas of the evaporator-superheater. The apparatus that absorbed the heat from the cooling system of the ASU compression stage was divided into heating, evaporating and overheating areas.

The results of the comparative analysis for the three schemes—without overheating and without internal recovery exchanger (Figure 4), without overheating and with internal recovery exchanger (Figure 5), and finally, the scheme with overheating and internal recovery exchanger (Figure 6)—are presented in Table 9.

The comparative analysis presented in Table 9 shows the decreasing trend of exergy destruction in the evaporator as the temperature of the ORC fluid increased and the temperature difference between the air to be cooled and the ORC fluid, which receives the heat, decreases. This result is also confirmed by studies [35,36].

The addition of the internal recuperative heat exchanger (IHE) was intended to increase the temperature of the ORC fluid at the inlet to the vaporizer; the maneuver proved to be correct because the share of relative exergy destruction in the heating area of the evaporator (ψ_h_) decreased (Table 9) from 4.8% to 3.743%, and decreased by almost a percentage, the overall relative destruction ($\psi_{ev}$) in the vaporizer.

In the case of the ORC scheme with the fluid overheating (Figure 6), the decrease in the exergy destruction caused by the heat transfer at the finite temperature difference between the air and the ORC fluid was more pronounced—the evaporation (ψ_zev_) and heating (ψ_h_) zones were discharged by transferring part of the destruction to the overheating area (ψ_oh_) (Table 9). Overall, the destruction of exergy associated with heat transfer in the evaporator-superheater decreased, reaching the lowest value compared with other schemes. Consequently, the value of the exergetic efficiency and the mechanical power produced were the highest for the scheme with overheating and internal recuperator changer.

### 5.2. Comparative Study of the Performance of the ORC Cycle Working with Different Organic Fluids

In the case of the organic fluid R245fa, the cycle with overheating and with internal recovery heat exchanger proved to be the most efficient. The performance of this scheme was also analyzed for other organic fluids with non-negative saturated vapor slopes, such as R123 and n-pentane.

From the category of “wet” fluids with a negative saturated vapor slope, NH_3_, a natural fluid, was chosen. In the case of NH_3_, the cycle was with expansion in the turbine up to the wet area, and consequently the internal recovery heat exchanger was missing.

#### 5.2.1. The Performance Study of the R717 (NH_3_) ORC with Overheating Using R717 (NH_3_)

**Figure 11:**
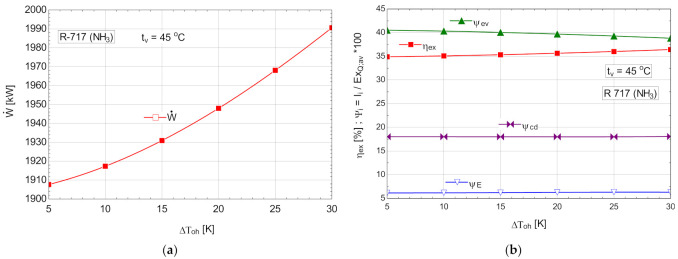
ORC with R-717 with overheating: (**a**) mechanical power, depending on the degree of overheating, $\Delta T_{oh}$; (**b**) exergetic efficiency and weights of exergy destructions relative to the available exergy of recovered heat, depending on the variation of the degree of overheating, $\Delta T_{oh}$.

The energetic and exergetic measures for the ORC cycle with R-717, with overheating are presented in Table 10 and Figure 11.

#### 5.2.2. ORC Cycle Performance Study with Internal Recovery Heat Exchanger and Overheating with R123 

The schematic of the system is shown in Figure 6.

**Figure 12:**
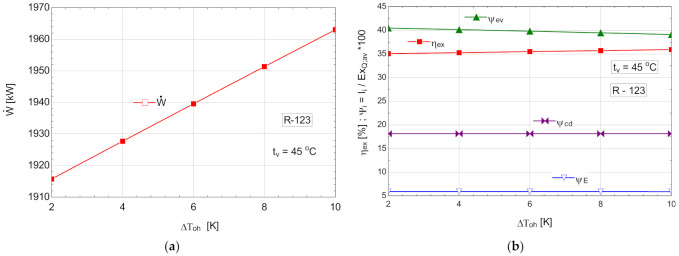
ORC with R-123 with internal heat exchanger and overheating: (**a**) mechanical power, depending on the degree of overheating, $\Delta T_{oh}$; (**b**) exergetic efficiency and weights of exergy destructions relative to the exergy of available recovered heat, depending on the variation of the degree of overheating, $\Delta T_{oh}$.

The results of the analysis are presented in Table 11 and Figure 12.

#### 5.2.3. Performance Study for the ORC with Internal Recovery Heat Exchanger and Overheating, Operating with n-Pentane 

The schematic of the system is shown in Figure 6.

**Figure 13:**
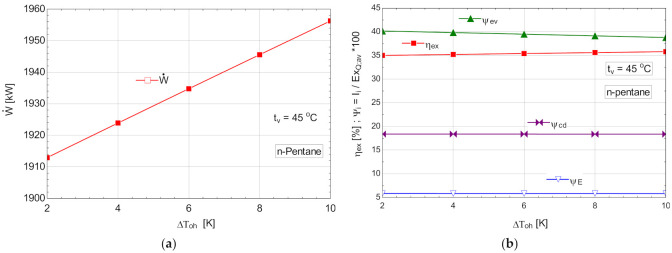
ORC with n-pentane with internal heat exchanger and overheating; (**a**) mechanical power, depending on the degree of overheating, $\Delta T_{oh}$; (**b**) exergetic efficiency and weights of exergy destructions relative to the exergy of available recovered heat, depending on the variation of the degree of overheating, $\Delta T_{oh}$.

The results of the analysis are presented in Table 12 and Figure 13.

#### 5.2.4. Comparative Results on the Performance of Rankine Cycles Working with Different Organic Fluids

Under the conditions imposed by the recovery of the heat evacuated by the compression area of the cryogenic air separation unit and following the comparative study using different organic fluids, the following resulted (Table 13).

The final choice for the working fluid was conducted on a risk and economic analysis.

When using the mechanical energy produced in the expander $\dot{W}=1991\;kW$ (Table 13) the electrical energy used for driving the compressors decreased by 6% compared with the initial value ${\dot{E}}_{el}=31755\;kW$ (Table 1).

(28) $$\Delta {\dot{E}}_{el}=\frac{\dot{W}}{{\dot{E}}_{el}}=\frac{1991}{31755}=0.06$$

## 6. Conclusions

The exergetic analysis of the cryogenic air separation unit revealed an exergetic efficiency of the global system of 14%, and a share of the exergy loss with heat evacuated by the compressor cooling system of 21% of the total electricity consumption necessary to drive the compressors.

The loss of exergy in the compression area suggested, as a measure to increase the performance of the installation, the recovery of the heat transferred to the cooling system of the compressors and its transformation into mechanical energy with the help of ORC cycles.

To reduce the exergy destruction in the heat exchanger through which the ORC fluid absorbed heat from the compressed air stream (the ORC evaporator), an attempt was conducted to reduce the temperature difference between the air and the ORC fluid by increasing the temperature of the ORC fluid at the inlet to the heat exchanger. In this sense, the scheme of the ORC installation was structurally modified by adding an internal recuperative heat exchanger; the effect was to reduce the overall exergy destruction in the heat exchanger by decreasing the exergy destruction in the heating zone of the ORC fluid until the saturation temperature (vaporization temperature) was reached and the mechanical power produced was increased accordingly.

To further reduce the destruction of exergy caused by the large temperature difference between the compressed air and the ORC fluid, the organic fluid vapor was overheated. As expected, the perfection of the cycle represented by its exergetic efficiency improved. The effect of increasing the enthalpy fall in the expander due to overheating was tempered by the decrease in the mass flow rate, but overall, the produced mechanical power increased.

The conclusions drawn from the exergetic analysis of the behavior of the ORC cycle with R-245fa were extended to other organic fluids both in the category of those with a non-negative saturated vapor slope, and those in which the expansion in the expander reached the wetland. Investigations were conducted, based on an exergetic analysis of the operation of the cycle, as to which achieved the best results with R245fa (cycle with overheating and internal recuperative heat exchanger) in the case of operation with R123, n-pentane and R717.

For all organic agents, the increase in the vaporization temperature obviously led to an increase in the power produced by the ORC system. Unfortunately, the maximum value of the vaporization temperature was limited by the need to ensure a minimum temperature difference ($\Delta T_{P})\;$ in the evaporator of the installation. The same issue occurred with the degree of overheating for a given evaporation temperature.

If R717 was used as a working agent, due to the expansion of the gas in the expander to the wet area, the internal recovery heat exchanger was missing from the diagram; this disadvantage was offset by the possibility of operating with a much higher degree of overheating than in the case of the other fluids studied, which had a non-negative saturated vapor curve slope.

Among the cases studied, the variant with R717 and with overheating $\Delta T_{oh}=30_{}K$ achieved the highest mechanical power recovered from the heat evacuated by the compressed air, and the lowest mass flow rate of working fluid in the conditions of the best exergetic efficiency.

N-pentane was distinguished by low fluid flow and high mechanical power.

R123 had a much higher power than n-pentane but for a much higher organic fluid flow.

In addition to the thermodynamic analysis, a risk and economic analysis will tip the balance of the decision towards one of the analyzed ORCs working fluids.

Recovery of the heat evacuated when cooling the air in the compression area of the cryogenic air separation unit on the platform of the Galati Iron and Steel Works with the help of an ORC and its transformation into electricity is a viable solution. This can reduce the total electricity consumption for compressor drive by 6%.

## Figures and Tables

**Table 1:** Compressors input powers [26].

Compressor	${\left|\dot{W}\right|}_{}^{}\left[kW\right]$
1	10,292
2	10,712
3	10,751
**Global fuel of the installation F**	**31,755**

**Table 2:** Values of the exergetic products [26].

Substance	$\dot{E}x_{}^{TM}\left[kW\right]$	$\dot{E}x_{}^{CH}\left[kW\right]$	$\dot{E}x_{}^{TM}\left[kW\right]$
GO_2_	377.4	2752	3129.4
GN_2_	279	835.8	1114.8
LAr	151.5	89.53	241
**Global installation product P**			**4485.2**

**Table 3:** Exergy destruction in the compression stages [26].

Compressor Stage	$\mathrm{Destruction}\;\mathrm{of}\;\mathrm{Exergy}\;{\dot{I}}_{cp}\left[MW\right]$
1	1.659
2	1.647
3	1.652
$\mathrm{Total}\;{\dot{I}}_{cp}$	**4.958**

**Table 4:** Losses with heat exergies evacuated in the coolers of the compression stages [26].

Cooler	$\mathrm{Exergy}\;\mathrm{Heat}\;\mathrm{Loss}\;\left|\dot{E}x_{Q}\right|+{\dot{I}}_{\Delta p}\left[MW\right]$
1	2.3
2	2.32
3	2.161
$\mathrm{Total}\;{\dot{L}}_{Q}$	**6.781**

**Table 5:** The potential for recovery of the heat discharged by the compression stage of the ASU.

Heat Exchanger	$\mathrm{Air}\;\mathrm{Mass}\;\mathrm{Flow}\;\mathrm{Rate}{\dot{m}}_{a}\;[\mathrm{kg}/s]$	$\mathrm{Air}\;\mathrm{Inlet}\;\mathrm{Temperature}\;t_{i}$ °C	$\mathrm{Air}\;\mathrm{Outlet}\;\mathrm{Temperature}\;t_{o}$ °C	Thermal Power ${\dot{Q}}_{}\left[MW\right]$
Intermediate cooler 1	130.5	103.4	40	8.35
Intermediate cooler 2	130.5	121.5	40	10.76
Final cooler	130.5	121.8	30	12.16
$\mathrm{Total}\;\mathrm{available}\;\mathrm{thermal}\;\mathrm{power}\;\left({\dot{Q}}_{av}\right)$				**31.27**

**Table 6:** Energetic and exergetic measures for the ORC cycle with R-245fa, without overheating and without internal heat exchanger.

$t_{ev}$	$\eta_{ex}$	$\dot{W}$	${\dot{W}}_{E}$	${\dot{W}}_{P}$	${\dot{m}}_{ORC}$	$\psi_{ev}$	$\psi_{cd}$	$\psi_{E}$	$t_{2}$	$t_{5}$
$\left[{}^{\circ }C\right]$	[%]	[kW]	[kW]	[kW]	[kg/s]	[%]	[%]	[%]	$\left[{}^{\circ }C\right]$	$\left[{}^{\circ }C\right]$
35	21.44	1172	1187.82	15.82	146.9	56.4	18.36	3.675	23.09	15.06
38	25.25	1380	1400	20	145.4	52.02	18.26	4.324	23.78	15.08
41	28.92	1580	1604	24	143.9	47.81	18.16	4.946	24.49	15.09
44	32.45	1773	1801.56	28.56	142.5	43.74	18.07	5.543	25.21	15.11
47	35.85	1959	1991.55	32.55	141.1	39.82	17.98	6.116	25.95	15.13
50	39.12	2138	2175.18	37.18	139.8	36.04	17.91	6.666	26.7	15.15

**Table 7:** Energetic and exergetic measures for the ORC with R-245fa, without overheating but with internal heat exchanger.

$t_{ev}$	$\eta_{ex}$	$\dot{W}$	${\dot{m}}_{ORC}$	$\psi_{ev}$	$\psi_{cd}$	$\psi_{E}$	$t_{2}$	$t_{5}$	$t_{7}$	$t_{s}$
$\left[{}^{\circ }C\right]$	[%]	[kW]	[kg/s]	[%]	[%]	[%]	$\left[{}^{\circ }C\right]$	$\left[{}^{\circ }C\right]$	$\left[{}^{\circ }C\right]$	$\left[{}^{\circ }C\right]$
35	21.7	1186	148.6	56.03	18.31	3.719	23.09	15.06	20.46	16.95
38	25.62	1400	147.5	51.51	18.18	4.386	23.78	15.08	20.57	17.38
41	29.41	1607	146.4	47.14	18.06	5.029	24.49	15.09	20.67	17.83
44	33.08	1808	145.3	42.9	17.93	5.651	25.21	15.11	20.78	18.29
47	36.64	2002	144.3	38.79	17.81	6.251	25.95	15.13	20.89	18.75
50	40.08	2190	143.2	34.8	17.7	6.831	26.7	15.15	21	19.23

**Table 8:** Energetic and exergetic measures for the ORC with R-245fa, with overheating and with internal heat exchanger, $t_{ev}=45\;{}^{\circ }C$.

ΔT_oh_	$\eta_{ex}$	$\dot{W}$	${\dot{m}}_{ORC}$	$\psi_{ev}$	$\psi_{cd}$	$\psi_{E}$	$t_{2}$	$t_{5}$	$t_{7}$	$t_{s}$
[K]	[%]	[kW]	[kg/s]	[%]	[%]	[%]	$\left[{}^{o}C\right]$	$\left[{}^{o}C\right]$	$\left[{}^{o}C\right]$	$\left[{}^{o}C\right]$
2	34.53	1887	144.7	41.15	17.89	5.855	27.53	15.12	21.13	19.7
4	34.78	1901	144.4	40.77	17.88	5.856	29.6	15.12	21.44	20.95
6	35.03	1914	144.1	40.39	17.88	5.857	31.66	15.12	21.75	22.2
8	35.27	1927	143.8	39.99	17.88	5.857	33.72	15.12	22.06	23.44
10	35.51	1940	143.6	39.59	17.87	5.856	35.77	15.12	22.37	24.68

**Table 9:** Comparative analysis: energetic and exergetic measures for different types of ORCs with R-245fa, ($t_{ev}=45\;{}^{\circ }C$ ).

**Cycle Type**	$\eta_{ex}$	$\dot{W}$	${\dot{m}}_{ORC}$	$\psi_{ev}$	$\psi_{h}$	$\psi_{zev}$	$\psi_{oh}$	$\psi_{cd}$	$\psi_{E}$
	[%]	[kW]	[kg/s]	[%]	[%]	[%]	[%]	[%]	[%]
Without IHE and without overheating	33.6	1836	142	42.42	4.803	37.61	-	18.04	5.737
With IHE and without overheating	34.28	1873	144.96	41.52	3.743	37.77	-	17.89	5.853
With IHE and overheating $\Delta T_{oh}=10K$	35.51	1940	143.6	39.59	1.916	34.4	3.265	17.87	5.856

**Table 10:** Energetic and exergetic measures for the ORC cycle with R-717, with overheating, $t_{ev}=45{}^{\circ }C$.

$\Delta T_{oh}$	$\eta_{ex}$	$\dot{W}$	${\dot{m}}_{ORC}$	$\psi_{ev}$	$\psi_{cd}$	$\psi_{E}$	$t_{2}$	$t_{5}$
[K]	[%]	[kW]	[kg/s]	[%]	[%]	[%]	$\left[{}^{o}C\right]$	$\left[{}^{o}C\right]$
5	34.91	1908	25.03	40.52	18.01	6.134	20	15.4
10	35.09	1917	24.69	40.33	18.01	6.161	20	15.4
15	35.34	1931	24.38	40.05	18	6.201	20	15.4
20	35.65	1948	24.08	39.7	17.99	6.252	20	15.4
25	36.02	1968	23.8	39.29	18	6.3	22.51	15.4
30	36.43	1991	23.53	38.62	18.07	6.29	26.92	15.4

**Table 11:** Energetic and exergetic measures for the ORC with R-123, with internal recovery exchanger and overheating, $t_{ev}=45\;{}^{\circ }C$.

$\Delta T_{oh}$	$\psi_{ex}$	$\dot{W}$	${\dot{m}}_{ORC}$	$\psi_{ev}$	$\psi_{cd}$	$\psi_{E}$	$t_{2}$	$t_{5}$	$t_{7}$	$t_{s}$
[K]	[%]	[kW]	[kg/s]	[%]	[%]	[%]	$\left[{}^{o}C\right]$	$\left[{}^{o}C\right]$	$\left[{}^{o}C\right]$	$\left[{}^{o}C\right]$
2	35.06	1916	161.5	40.48	18.14	5.897	26.92	15.08	21.04	19.11
4	35.28	1928	161.3	40.15	18.13	5.894	28.92	15.08	21.35	20.28
6	35.49	1940	161	39.81	18.13	5.89	30.92	15.08	21.65	21.45
8	35.71	1951	160.8	39.47	18.13	5.886	32.92	15.08	21.95	22.62
10	35.92	1963	160.5	39.11	18.13	5.882	34.91	15.08	22.26	23.79

**Table 12:** Energetic and exergetic measures for the n-pentane ORC cycle, with internal recovery exchanger and overheating, $t_{ev}=45\;{}^{\circ }C$.

$\Delta T_{oh}$	$\eta_{ex}$	$\dot{W}$	${\dot{m}}_{ORC}$	$\psi_{ev}$	$\psi_{cd}$	$\psi_{E}$	$t_{2}$	$t_{5}$	$t_{7}$	$t_{s}$
[K]	[%]	[kW]	[kg/s]	[%]	[%]	[%]	$\left[{}^{o}C\right]$	$\left[{}^{o}C\right]$	$\left[{}^{o}C\right]$	$\left[{}^{o}C\right]$
2	35.01	1913	75.58	40.16	18.38	5.842	30.08	15.06	21.53	21.43
4	35.21	1924	75.45	39.83	18.38	5.836	32.09	15.06	21.83	22.71
6	35.41	1935	75.32	39.49	18.38	5.83	34.11	15.06	22.14	23.99
8	35.6	1946	75.19	39.15	18.38	5.824	36.12	15.06	22.45	25.27
10	35.8	1956	75.06	38.79	18.37	5.818	38.13	15.06	22.77	26.55

**Table 13:** Performance parameters of ORC operating with different working fluids.

ORC Fluid	$\dot{W}$	${\dot{m}}_{ORC}$	$\eta_{ex}$	Functional Conditions
	[kW]	[kg/s]	[%]	
**R-245fa**	1940	143.6	35.51	ORC with overheating ΔT_oh_ = 10 K and internal heat exchanger, t_ev_ = 45 °C
**n-pentan**	1956	75.06	35.8	$\mathrm{ORC}\;\mathrm{with}\;\mathrm{overheating}\;\Delta T_{oh}=10K$ $\;\mathrm{and}\;\mathrm{internal}\;\mathrm{heat}\;\mathrm{exchanger},\;t_{ev}=45\;{}^{\circ }C$
**R-123**	1963	160.5	35.92	$\mathrm{ORC}\;\mathrm{with}\;\mathrm{overheating}\;\Delta T_{oh}=10K$ $\;\mathrm{and}\;\mathrm{internal}\;\mathrm{heat}\;\mathrm{exchanger},\;t_{ev}=45\;{}^{\circ }C$
**R717**	1991	23.53	36.43	$\mathrm{ORC}\;\mathrm{with}\;\mathrm{overheating},\;\Delta T_{oh}=30K$ $\;\mathrm{and}\;\mathrm{without}\;\mathrm{internal}\;\mathrm{heat}\;\mathrm{exchanger},\;t_{ev}=45\;{}^{\circ }C$

## Nomenclature

Cp	compressor
E	expander
${\dot{E}}_{el}$	current of electrical energy (exergy), kW
$\dot{E}x$	current of exergy, kW
Ev	evaporator
F	exergetic fuel, kW
$GN_{2}$	current of gaseous nitrogen, kmol/s
$GO_{2}$	current of gaseous oxygen, kmol/s
LAr	current of liquid argon, kmol/s
$\dot{Q}$	current of heat, kW
P	pump, exergetic product, kW
$\dot{W}$	mechanical power, kW
$\dot{E}x^{TM}$	current of thermo-mechanical exergy, kW
$\dot{E}x^{CH}$	current of chemical exergy, kW
h	mass enthalpy, kJ/kg
$\dot{I}$	current of exergy destruction due to internal irreversibility, kW
L	current of exergy loss, kW
$\dot{m}$	mass flow rate, kg/s
p	pressure, Pa
s	mass entropy, kJ/(kg K)
T	temperature, K
Subscripts
0	environment, in equilibrium with the environment
a	air
av	available
c	condensation
Cd	condenser
cp	compressor
ev	evaporator
ex	exergetic
i	inlet
h	heating of the liquid in the evaporator (Table 9)
L	loss
o	outlet
oh	overheating
ORC	ORC fluid
P	pump, pinch
Q	heat
zev	evaporation zone in the evaporator (Table 9)
w	water
Superscript
Cd	condenser
CH	chemical
T	thermodynamic temperature, K
TM	thermo-mechanical
TOT	total
Greek Symbols
ε	heat exchanger efficiency
Ψ	share of an exergetic loss or destruction in the fuel consumption
